# Revisiting the Electron Transfer Mechanisms in Ru(III)-Mediated Advanced Oxidation Processes with Peroxyacids and Ferrate(VI)

**DOI:** 10.1021/acs.est.4c02640

**Published:** 2024-06-20

**Authors:** Krishnamoorthy Sathiyan, Junyue Wang, Lois M. Williams, Ching-Hua Huang, Virender K. Sharma

**Affiliations:** † Program for Environment and Sustainability, Department of Environmental and Occupational Health, School of Public Health, Texas A&M University, College Station, Texas 77843-8371, United States; ‡ School of Civil and Environmental Engineering, Georgia Institute of Technology, Atlanta, Georgia 30332, United States

**Keywords:** peroxyacids (POAs), ferrate(VI), advanced oxidation processes (AOPs), ruthenium

## Abstract

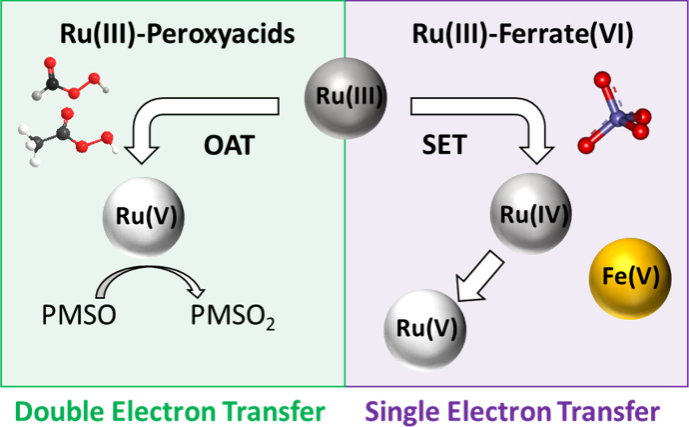

The potential of Ru(III)-mediated advanced oxidation processes has attracted attention due to the recyclable catalysis, high efficiency at circumneutral pHs, and robust resistance against background anions (e.g., phosphate). However, the reactive species in Ru(III)-peracetic acid (PAA) and Ru(III)-ferrate(VI) (FeO_4_^2–^) systems have not been rigorously examined and were tentatively attributed to organic radicals (CH_3_C(O)O^•^/CH_3_C(O)OO^•^) and Fe(IV)/Ru(V), representing single electron transfer (SET) and double electron transfer (DET) mechanisms, respectively. Herein, the reaction mechanisms of both systems were investigated by chemical probes, stoichiometry, and electrochemical analysis, revealing different reaction pathways. The negligible contribution of hydroxyl (HO^•^) and organic (CH_3_C(O)O^•^/CH_3_C(O)OO^•^) radicals in the Ru(III)-PAA system clearly indicated a DET reaction via oxygen atom transfer (OAT) that produces Ru(V) as the only reactive species. Further, the Ru(III)-performic acid (PFA) system exhibited a similar OAT oxidation mechanism and efficiency. In contrast, the 1:2 stoichiometry and negligible Fe(IV) formation suggested the SET reaction between Ru(III) and ferrate(VI), generating Ru(IV), Ru(V), and Fe(V) as reactive species for micropollutant abatement. Despite the slower oxidation rate constant (kinetically modeled), Ru(V) could contribute comparably as Fe(V) to oxidation due to its higher steady-state concentration. These reaction mechanisms are distinctly different from the previous studies and provide new mechanistic insights into Ru chemistry and Ru(III)-based AOPs.

## Abstract 2

New systematic investigation of this work identifies the mechanisms for Ru(III) activation of peroxyacids and ferrate(VI) in advanced oxidation processes to be oxygen atom transfer and single electron transfer reactions, respectively.

## Introduction

Organic micropollutants, including pesticides, pharmaceuticals, personal care products, and other chemical additives, have posed substantial threats to eco-sustainability and public health.^1,2^ Over the past few decades, numerous innovative oxidation technologies have been developed to eliminate organic micropollutants from wastewater, drinking water, and natural environments. In particular, metal-based advanced oxidation processes (AOPs) are a group of emerging oxidation technologies that attract substantial research due to their high efficiency in degrading micropollutants. Metal-based AOPs apply aqueous metal ions, metal-containing minerals,^3^ or single metal atom catalysts^4−6^ to activate oxidants and generate highly reactive high-valent metals or nonmetal radicals. The metal activators may react with the oxidants through either single electron transfer (SET) or double electron transfer (DET).^7−10^ The SET reactions lead to the formation of both high-valent metal species and radicals, while DET reactions are usually based on oxygen atom transfer (OAT) from oxidants to metals and generate only high-valent metals as the reactive species. Compared with radicals, high-valent metals generally have a higher steady-state concentration and better selectivity toward micropollutants due to targeting their electron-rich moieties.^11−13^ Furthermore, high-valent metal species may resist the influence of matrices in the treatment of real water samples. High-valent iron (Fe(IV)/Fe(V), ferrate(VI) (Fe^VI^O_4_^2–^)) species usually selectively react with contaminants with phenolic,^14−16^ nitrogen-containing,^17−20^ or sulfur-containing functional groups^18,21^ while exhibit low reactivity toward merely aliphatic or aromatic compounds. For instance, benzoic acid, a commonly used probe compound and typical structure in natural organic matter, is inert to high-valent iron but susceptible to oxidation by nonmetallic radicals, including HO^•^, SO_4_^•–^, and Cl^•^.^10,22,23^ In addition, unlike radicals susceptible to scavenging by halides,^24,25^ ferrate(VI) exhibits relatively low reactivity to bromide and almost no reactivity to chloride, hence mitigating the formation of toxic halogenated byproducts.^22,26−28^

Ruthenium (Ru)-based catalysts have been extensively investigated for electrochemical systems for carbon dioxide reduction^29^ and water splitting.^30^ Recently, Ru(III) and Ru-based materials, in combination with various oxidants (e.g., permanganate,^31^ periodate,^32^ peracetic acid (PAA),^33^ ferrate(VI)^34^), also emerge as promising metal activators in water decontamination AOPs.^35^ Compared with other Fenton-like AOPs, Ru(III)-based systems exhibit several remarkable advantages: (i) Ru-AOPs usually achieve satisfactory oxidant efficiency at circumneutral pHs,^32−34^ overcoming the pH restriction of other Fenton-like AOPs (e.g., Fe-AOPs)^9,22,36−40^; (ii) Ru-AOPs resist the inhibition effect of phosphate buffer that commonly observed in Fe- and Co-AOPs due to complexation with metals^37,41,42^; and (iii) unlike Fe- and Mn-AOPs that are easily terminated by the formation of inactive Fe(III) and Mn(IV) species,^36,43^ Ru(III) could be recycled in the AOP systems because active Ru(III) could be reformed during Ru(IV)/Ru(V) oxidation of micropollutants.^32^ These recent studies are of great value and open opportunities for Ru-based catalytic water decontamination.

However, the reaction mechanisms of Ru(III) with oxidants are always intuitively proposed without rigorous examination, hindering a systematic understanding of the Ru chemistry and the potential of Ru-AOPs in different water matrices. For example, the oxidation capacity of Ru(III)-PAA was attributed to organic radicals (CH_3_C(O)O^•^/CH_3_C(O)OO^•^) generated by SET between PAA and Ru species, while no probe was used to differentiate organic radicals and high-valent Ru species.^33^ An innovative Ru(III)-ferrate(VI) AOP has been reported; however, the DET reaction between ferrate(VI) and Ru(III) was proposed with merely density functional theory (DFT) calculations rather than experimental evidence.^34^

Therefore, the objective of this study was to systematically reinvestigate the reaction pathways (SET vs DET/OAT) between Ru(III) and PAA and ferrate(VI). In addition, we also introduced and mechanistically evaluated the Ru(III) activation of performic acid (PFA) for the first time. PFA is another peroxyacid (POA) receiving increasing attention,^44−47^ in comparison to PAA. Chemical probes, quenchers, stoichiometry, and electrochemistry analysis were applied to reveal reaction pathways different from those suggested by previous studies, which provides new insights and methodologies for studying electron transfer mechanisms in metal-AOPs.

## Materials and Methods

### Chemicals

Performic acid (PFA)^48^ and potassium ferrate(VI) (K_2_FeO_4_)^49^ were synthesized in our laboratories according to reported protocols. PAA solution (32% PAA and 6% H_2_O_2_ w/w in acetic acid and water solution) and hydrogen peroxide solution (30% H_2_O_2_ w/w in water) were purchased from Sigma-Aldrich (St. Louis, MO). The stock solutions of Ru(III) and oxidants were prepared freshly before each experiment in DI water (or 10 mM borate buffer (pH 9.0) for ferrate(VI)). The concentration of ferrate(VI) was determined using a UV–vis spectrophotometer (DR-5000, Hach 48 Co., USA) by measuring the absorbance at 510 nm, (ε = 1150 M^–1^ cm^–1^), and titration methods determined the concentrations of PAA and H_2_O_2_ solutions.^36^ A list of other used chemicals is provided in Text S1.

### Batch Experiments for Micropollutant Degradation

The experiments were carried out in 50 mL beakers with constant magnetic stirring. The solutions contained Ru(III) and micropollutants at designed concentrations, and the pH was adjusted to desired values by (i) adding sodium hydroxide and/or sulfuric acid or (ii) employing buffer (phosphate for PFA, PAA, H_2_O_2_, borate for ferrate(VI)). Then, an oxidant was added to initiate the reactions.

Periodically, 1.0 mL samples were collected and quenched with 10 mM thiosulfate (Na_2_S_2_O_3_, for POAs) or hydroxylamine (for ferrate(VI)). The micropollutant concentrations were measured by high-performance liquid chromatography equipped with an Agilent Zorbax SB-C18 column (2.1 × 150 mm, 5 μm) and a diode array detector (HPLC-DAD) with detailed methods reported in our previous studies.^14,43^ Briefly, acetonitrile and water were used as the eluents at flow rates of 0.2–0.4 mL/min, and the compounds were analyzed based on their absorbance at 210–270 nm.

### UV–Visible Spectrophotometry

To analyze ferrate(VI) and Fe(IV) species in the system, the absorbance spectra of the mixture solution were scanned in the range 200–500 nm by a UV–visible spectrophotometer. Ferrate(VI) was identified based on its absorption peak at 510 nm. Note that, although Ru(III) and Ru(V) have absorbance at 510 nm, their spectra do not exhibit a peak shape near 510 nm.^32^ Thus, the exact concentration of ferrate(VI) could not be quantified by absorbance at 510 nm; rather, we use the disappearance of characteristic spectra of Fe(VI) to indicate the ferrate(VI) consumption. Fe(IV) was measured by reducing it to Fe(II) by methyl phenyl sulfoxide (PMSO) and monitoring Fe(II) by the 2,2′-bipyridine complexation method (see later discussion).

### Electrochemical Measurements

Differential pulse voltammetry (DPV) was performed to identify the formation of high-valent Ru species. A three-electrode system (CH Instrument electrochemical workstation) was applied to perform the electrochemical measurements. A 3 mm glassy carbon electrode acted as the working electrode, and graphite rod and Hg/HgO electrodes were used as the counter electrode and reference electrode, respectively. The working electrode was first polished with 0.30 μm Al_2_O_3_ powder to remove any impurities, followed by polishing with 0.05 μm Al_2_O_3_ powder to ensure a smooth surface. The DPV analysis was conducted with the following parameters: amplitude = 50 mV, pulse width = 0.05 s, and pulse period = 0.2 s. All of the potentials mentioned in this work are referenced to the reversible hydrogen electrode (RHE) (eq 1).
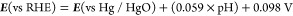
1

## Results and Discussion

### Oxidation Performance of Ru(III)-POAs

**Figure 1:**
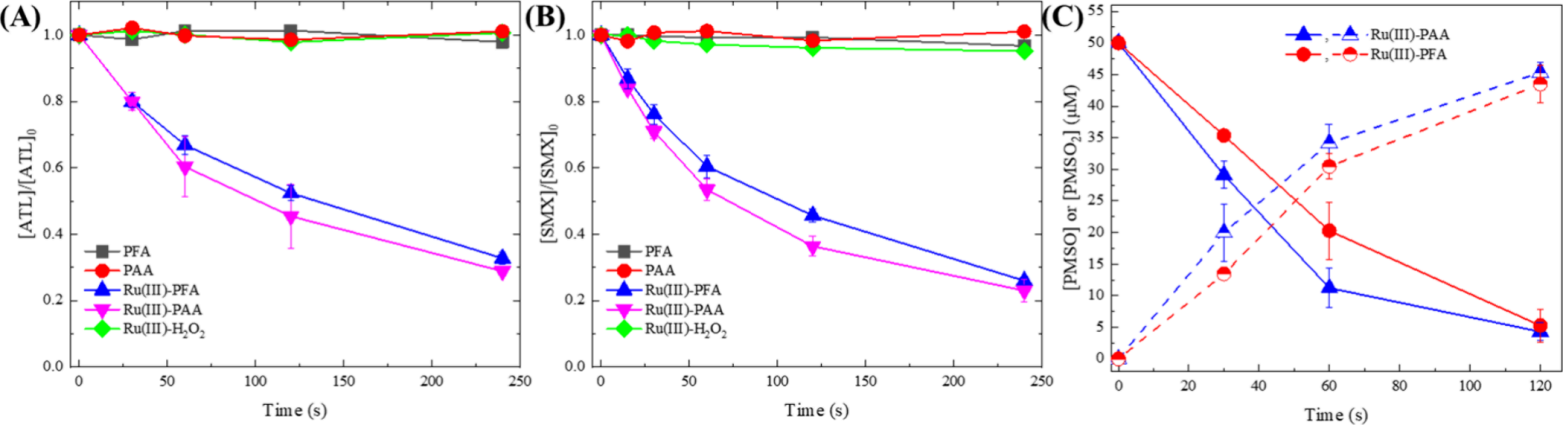
Degradation of ATL (A) and SMX (B), and PMSO (C) by POAs and Ru(III)-POA processes. Experimental conditions: [ATL]_0_ = [SMX]_0_ = 5 μM, [PMSO]_0_ = 50 μM, [POAs]_0_ = [H_2_O_2_]_0_ = [Ru(III)]_0_ = 100 μM, pH = 7.0, [phosphate buffer] = 10 mM.

The oxidation capacity of Ru(III)-POA processes was evaluated by degrading two commonly detected pharmaceuticals: atenolol (ATL) and sulfamethoxazole (SMX). The experiments were conducted at pH 7.0, the optimal pH for Ru(III)-PAA,^33^ where 94.3% of PAA (p*K*a = 8.2^50^) and 66.7% PFA (p*K*a = 7.3^51^) were protonated. As shown in Figure 1A,B, POAs themselves were not able to degrade ATL or SMX within 4.0 min at pH 7.0. These results are consistent with previous studies that POAs only selectively react with thiols, thioethers, and tertiary amines, while they are unable to oxidize other organic compounds effectively.^44,52,53^ Notably, although 100 μM PFA and PAA solutions contained 60 and 40 μM of H_2_O_2_, respectively, due to the synthesis procedure, the coexistent H_2_O_2_ contributed negligibly to the oxidation processes (Figure 1A,B). However, Ru(III)-activated POA resulted in >60% removal of both pharmaceuticals within 4 min. The oxidation of the contaminants by Ru(III)-PFA was slightly slower than that by Ru(III)-PAA, but comparable.

The oxidation capacity of Ru(III)-PAA was already reported in our previous study^33^; hence, the primary objective of this study is to re-examine the oxidation mechanisms and reactive species (see further discussion). Compared with PAA, PFA is a less stable oxidant due to spontaneous decay but disinfects microorganisms with higher capacity.^45,47,48,53^ However, the potential of PFA in AOPs has never been explored. This study represents the first demonstration of PFA in AOPs, and the potential of other PFA-AOPs will be explored in the future.

### Reactive Species and Reaction Pathways of Ru(III)-POAs

The reactions between Ru(III) and POAs may proceed via (1) SET generating Ru(IV), carboxylic acids and hydroxyl radical (HO^•^) (eq 2), (2) SET generating Ru(IV) and acyloxyl radical (eq 3), or (3) DET generating Ru(V) and carboxylic acids (eq 4), rendering HO^•^, Ru(IV)/Ru(V) as candidate reactive species for the oxidation of ATL and SMX. In addition, HO^•^ and high-valent Ru (i.e., Ru(IV)/Ru(V)), if generated, may also oxidize POAs to produce acylperoxyl radicals (eq 5), which have been reported to exhibit considerable oxidation capacity toward structurally diverse organic compounds and pathogens.^54−56^
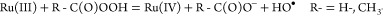
2
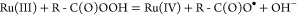
3
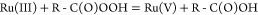
4
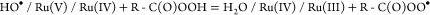
5

Notably, the organic radicals in PFA/PAA systems include acetyl(per)oxyl radicals (CH_3_C(O)O^•^/CH_3_C(O)OO^•^) and formyl(per)oxyl radicals (HC(O)O^•^/HC(O)OO^•^), which exhibit distinctly different chemical properties and require separate discussion. CH_3_C(O)OO^•^ is among the most reactive organic radical and has a relatively slow self-decay rate (eq 6), which could contribute to oxidation processes.^56,57^ However, the other three radicals usually undergo rapid decay and hardly contribute to oxidation. CH_3_C(O)O^•^ decomposes via a decarboxylation pathway (eq 7) to produce ^•^CH_3_ with low reactivity.^56^ HC(O)O^•^ undergoes rapid rearrangement to produce ^•^COO^–^, which both react with oxygen to give superoxide radical (HO_2_^•^/O_2_^•–^) (eq 8),^58^ while HC(O)OO^•^ is also easily hydrated to produce HO_2_^•^/O_2_^•–^ (eq 9).^58,59^ The produced superoxide radicals also exhibit low reactivity and usually contribute negligibly in AOPs.^60−62^
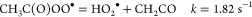
6
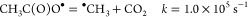
7
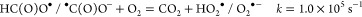
8
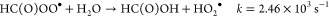
9

**Figure 2:**
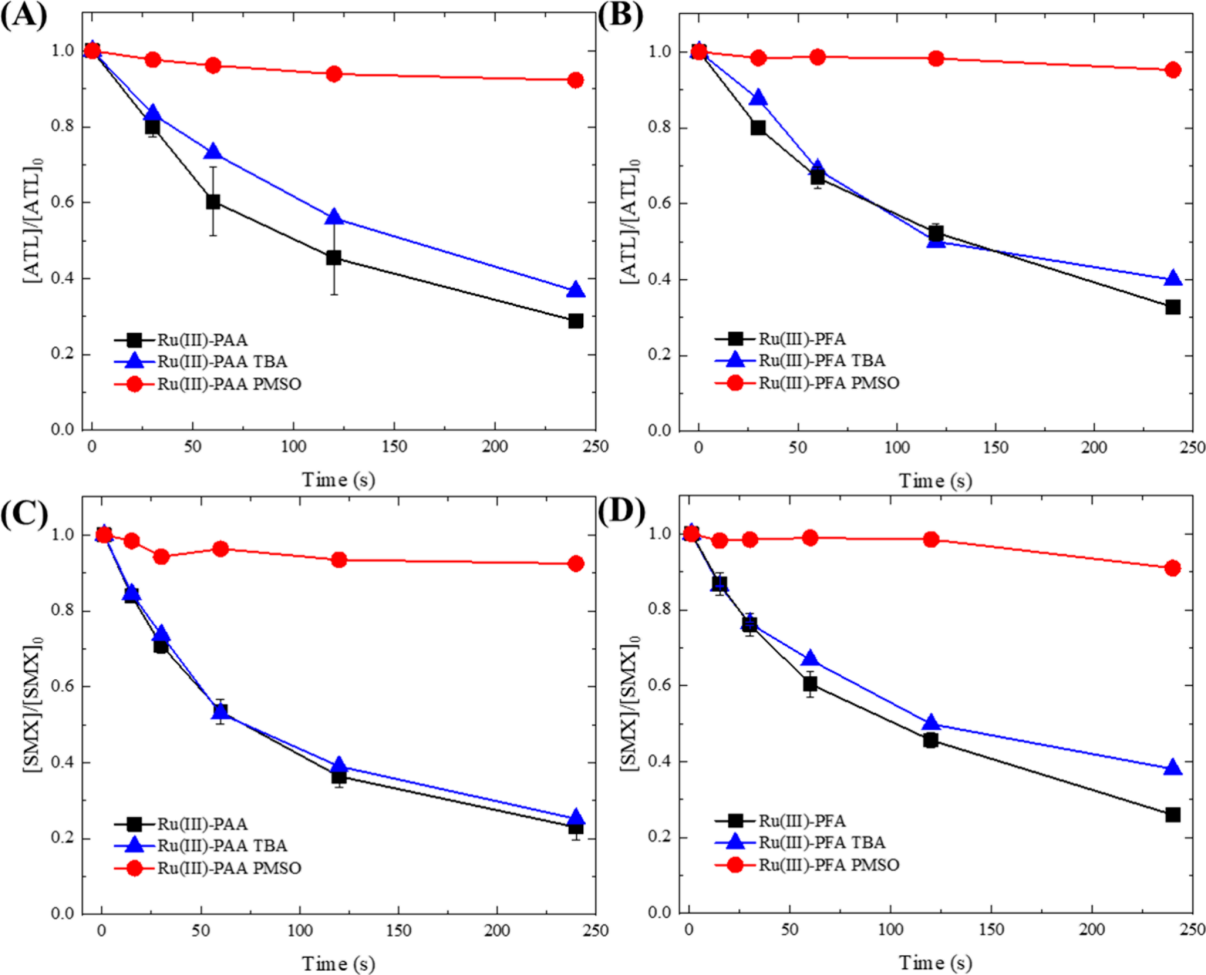
Degradation of ATL (A, B) and SMX (C, D) by Ru(III)-POA processes with quenchers. Experimental conditions: [ATL]_0_ = [SMX]_0_ = 5 μM, [TBA]_0_ = 10 mM, [PMSO]_0_ = 1 mM, [POAs]_0_ = [Ru(III)]_0_ = 100 μM, pH = 7.0, [phosphate buffer] = 10 mM.

First, the role of high-valent Ru and HO^•^ was evaluated by the oxidation of PMSO. It is well documented that Ru(V) selectively oxidizes PMSO to methyl phenyl sulfone (PMSO_2_) with a 100% conversion,^32^ while HO^•^ efficiently oxidizes PMSO to other products.^22^ As shown in Figure 1C, we found that PMSO was selectively converted to PMSO_2_ by both Ru(III)-PFA and Ru(III)-PAA processes, with a near 100% yield, indicating the critical role of Ru(V) (eq 4) and a negligible contribution of HO^•^. Furthermore, the addition of *tert*-butyl alcohol (TBA), a selective quencher for HO^•^, hardly affected the degradation of ATL and SMX by Ru(III)-POAs (Figure 2), further confirming the negligible role of HO^•^ and ruling out eq 2 as a major reaction pathway. However, whether the organic radical-producing SET (eq 3) can co-occur with the Ru(V)-generating DET reaction (eq 4) remains unclear based on these experiments.

**Figure 3:**
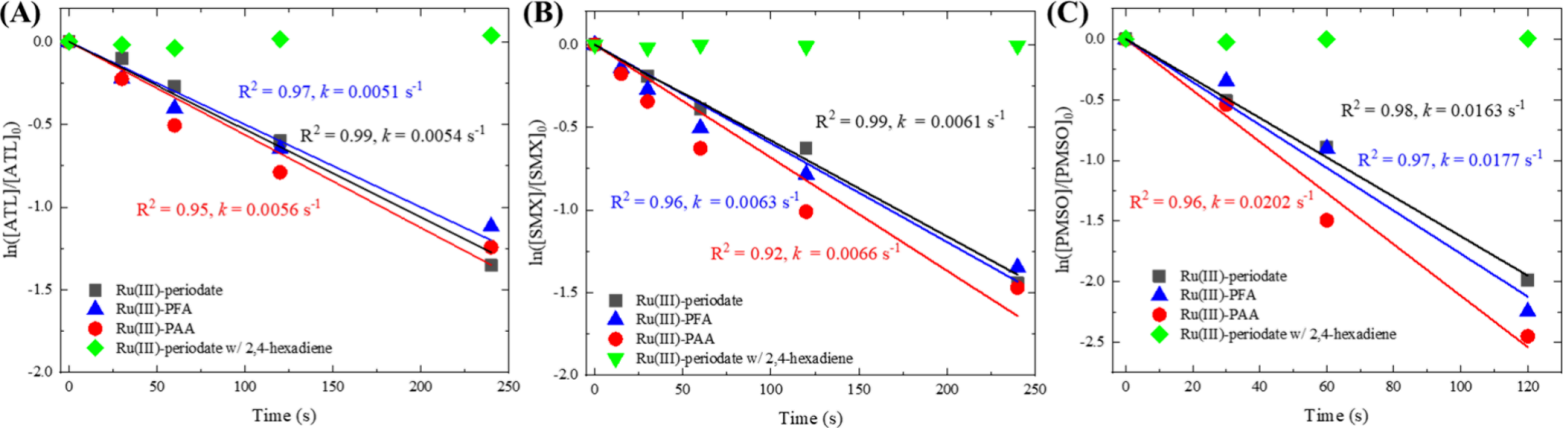
Degradation of ATL (A) and SMX (B), and PMSO (C) by Ru(III)-POA and Ru(III)-periodate processes. Experimental conditions: [ATL]_0_ = [SMX]_0_ = 5 μM, [PMSO]_0_ = 50 μM, [POAs]_0_ = [periodate]_0_ = [Ru(III)]_0_ = 100 μM, [2,4-hexadiene]_0_ = 0.5 mM, pH = 7.0, [phosphate buffer] = 10 mM. The solid lines represent linear regression of ln *C*/*C*_0_ values, and the *k* values are pseudo-first-order degradation rate constants.

Therefore, the contribution of acetyl(per)oxyl radicals (CH_3_C(O)O^•^/CH_3_C(O)OO^•^) generated by eqs 3 and 5 in Ru(III)-PAA was further evaluated. As reported in our previous study, CH_3_C(O)O^•^/CH_3_C(O)OO^•^ exhibit sluggish reactivity toward PMSO and do not yield PMSO_2_ as a transformation product.^41^ Thus, considering that CH_3_C(O)O^•^/CH_3_C(O)OO^•^ may be generated while contributing negligibly to PMSO oxidation, the ∼100% PMSO_2_ yield could not unequivocally prove their absence in the Ru(III)-PAA system. Therefore, we further applied PMSO as a quencher for ATL and SMX oxidations (Figure 2). If CH_3_C(O)O^•^/CH_3_C(O)OO^•^ were inert to PMSO, their contribution to ATL or SMX degradation could not be entirely eliminated by the addition of 1 mM PMSO. Nonetheless, we found that 1 mM PMSO almost completely suppressed the degradation of ATL and SMX by Ru(III)-POA processes, indicating the negligible contribution of organic radicals in the system. To address the potential concerns on the reactivity of different organic radicals toward ATL and SMX, we further tested the degradation of naproxen (NPX), whose reactivity with CH_3_C(O)O^•^/CH_3_C(O)OO^•^ has been confirmed in multiple studies,^50,56^ by Ru(III)-POA processes. As shown in Figure S1, 1 mM of PMSO also completely inhibited the NPX degradation by Ru(III)-PAA and Ru(III)-PFA. As discussed, if any of CH_3_C(O)O^•^/CH_3_C(O)OO^•^ was generated, they should lead to NPX degradation not quenchable by PMSO. Thus, CH_3_C(O)O^•^/CH_3_C(O)OO^•^ was negligibly produced, and reactions 3 and 5 were not operative in Ru(III)-PAA. It should be noted that, although Li et al. reported that the oxidation by Ru(III)-PAA could be quenched by 2,4-hexadiene (a selective quencher for acyl(per)oxyl radicals),^33^ we found that 2,4-hexadiene also scavenged Ru(V), which is known to be produced in the Ru(III)-periodate process^32^ and evaluated by new results in this work (see Figure 3). Thus, the 2,4-hexadiene quenching experiments were not contradictory to the Ru(V)-producing pathway proposed in this study.

For the Ru(III)-PFA system, due to rapid self-decay of PFA (eqs 8 and 9), the formation of HC(O)O^•^/HC(O)OO^•^ through SET pathways (eqs 3 and 5) could not be directly ruled out, even if they did not contribute to oxidation of SMX/ATL (i.e., HC(O)O^•^/HC(O)OO^•^ might be produced and then undergo fast decay). For this, we assessed the Ru(V) generation efficiency of Ru(III)-periodate, Ru(III)-PAA, and Ru(III)-PFA processes by comparing their oxidation kinetics of micropollutants. Ru(III)-periodate generates Ru(V) rapidly (<5 s) with nearly 100% electron efficiency^32^; hence, 100 μM of equimolar Ru(III) and periodate produces ∼100 μM of Ru(V). As reported, 100 μM of Ru(III) could be rapidly oxidized to 100 uM of Ru(V) by periodate, and the generated Ru(V) is relatively stable in the solution.^32^ Therefore, the Ru(III)-periodate system contained ∼100 μM Ru(V), as the only oxidation contributor, throughout the 4 min oxidation of micropollutants. As any significant formation of HO^•^ (eq 2) and CH_3_C(O)O^•^/HC(O)O^•^ (eqs 3 and 5) in Ru(III)-POA was ruled out by TBA, PMSO, and NPX experiments, yet the Ru(III)-POA processes achieved similar or even slightly faster oxidation of micropollutants than Ru(III)-periodate (Figure 3), indicating that the electron efficiency for Ru(V) generation in Ru(III)-POA was also near 100%. Thus, the SET pathway should be insignificant in the Ru(III)-POA processes.

Thus far, we have demonstrated that Ru(V) was the only reactive species in the Ru(III)-POA systems. The negligible roles of HO^•^ and organic radicals indicated that the SET reactions proposed in earlier studies were in fact unimportant during the Ru(III)-POA interaction (reactions 3 and 5).^33^ Rather, Ru(III) reacts with POA via the DET reaction and generates Ru(V) as the predominant reactive species (eq 4).

### Oxidation Performance of Ru(III)-Ferrate(VI)

**Figure 4:**
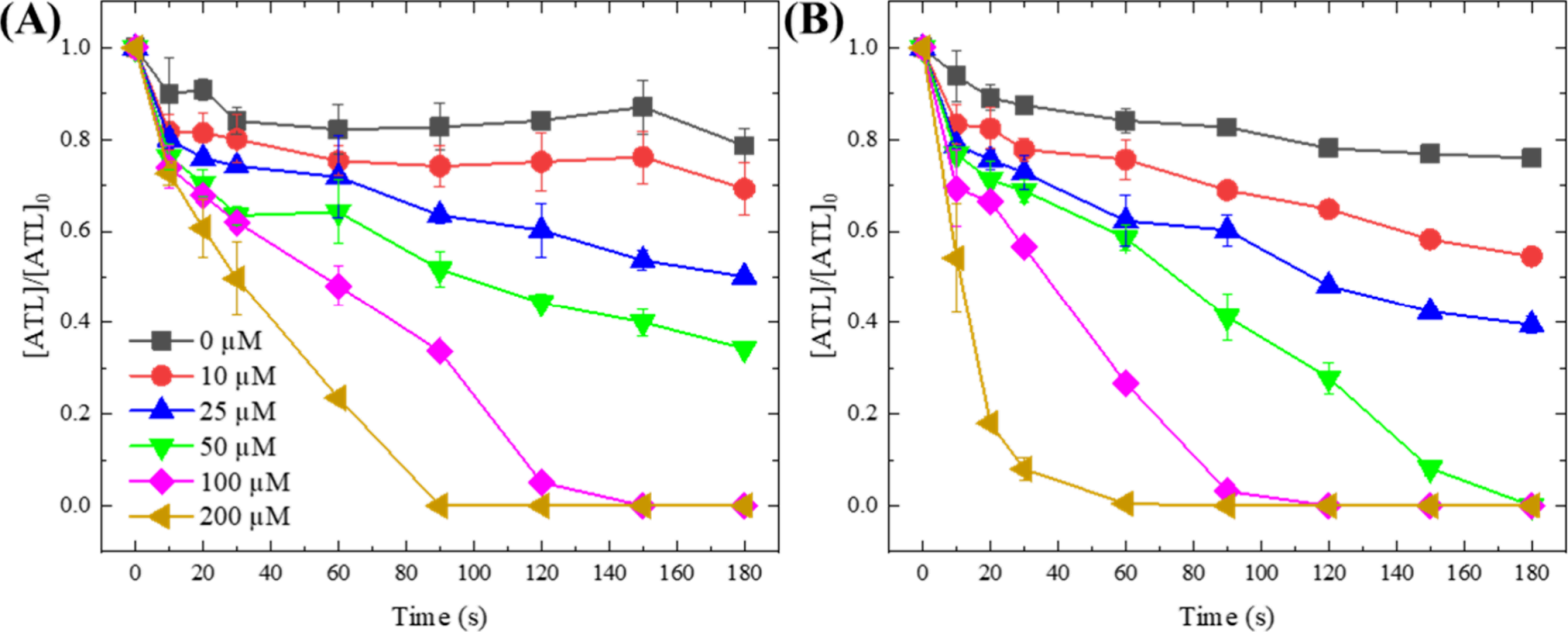
Degradation of ATL by Ru(III)-ferrate(VI) processes with different Ru(III) concentrations at pHs 9.0 (A) and 8.0 (B). Experimental conditions: [ATL]_0_ = 5 μM, [ferrate(VI)] = 100 μM, [borate buffer] = 10 mM.

We reinvestigated the reaction between Ru(III) and ferrate(VI). The degradation of ATL was sought by Ru(III)-ferrate(VI) with molar ratios ([Ru(III)]_0_:[ferrate(VI)]_0_) varied from 0.10 to 2.0 at pHs 9.0 and 8.0, where ferrate(VI) was relatively stable, and the oxidation could be measured.^63−65^ Concurring with previous studies, ferrate(VI) alone resulted in a mediocre oxidation efficiency at both pHs 9.0 and 8.0 (i.e., ∼20% removal after 3.0 min).^66,67^ As shown in Figure 4, the degradation of ATL increased significantly with an increasing Ru(III) dosages. At high molar ratios of 1.0, 1.5, and 2.0, complete degradation of ATL was observed at 150, 90, and 60 s, respectively. Interestingly, unlike the pseudo-first-order ATL degradation by Ru(III)-POA, the degradation of ATL by Ru(III)-ferrate(VI) consisted of two oxidation stages (Figure 4). Not surprisingly, the ATL degradation at pH 8.0 was faster than that at pH 9.0, particularly in the initial fast reaction stage. The initial stage was a rapid oxidation stage that finished in the first 10 s and contributed to 16.8–45.9% ATL degradation ([Ru(III)]_0_ = 10–200 μM, pH = 8.0), which was likely attributed to short-lived and highly reactive species. The second oxidation stage was slower and lasted for >3.0 min, which could be ascribed to the long-lasting oxidation by stable reactive species. These oxidation patterns suggest multiple oxidative species with different stability and reactivity reacted with ATL to cause complete degradation.

More experiments were conducted to evaluate the capability of Ru(III)-ferrate(VI) for removal of other micropollutants including SMX, carbamazepine (CBZ), trimethoprim (TMP), aspartame (APT), and diatrizoic acid (DTA) (Figure S2C). A molar ratio of 0.5 ([Ru(III)]_0_:[ferrate(VI)]_0_) was applied to seek their removal. Except for APT and DTA, the removal of other compounds was significantly enhanced with the addition of Ru(III), confirming the capability of Ru(III)-ferrate(VI) in degrading various contaminants with diverse moieties.

Ru(III) addition also significantly accelerated PMSO oxidation and PMSO_2_ formation by ferrate(VI). The participation of high-valent Ru and Fe species was evidenced in the near-complete conversion of PMSO to PMSO_2_ at different molar ratios from 0.10 to 0.50 (Figure S3). However, as both Ru(V) and Fe(IV)/Fe(V) could oxidize PMSO to PMSO_2_, PMSO oxidation could not be utilized to differentiate high-valent Ru and Fe species.^22,32,34^

### Reactive Species and Reaction Pathways of Ru(III)-Ferrate(VI)

The reaction between Ru(III) and ferrate(VI) may proceed via either SET (eqs 10 and 11) or DET (eq 12) reactions, generating either Fe(IV)/Fe(V) and Ru(IV)/Ru(V) as the reactive species to oxidize ATL. Fe(V), once generated by SET, undergoes rapid unimolecular and bimolecular self-decay (eqs 13 and 14).^65^ In particular, the first-order decay has a considerable rate constant ranging from 5.0 (pH 9.0) to 100 s^–1^ (pH 8.0), which leads to the rapid formation of inactive Fe(III) and could hardly contribute to further Ru(III) oxidation. Therefore, the SET mechanism likely results in a 1:2 overall stoichiometry between Ru(III) and ferrate(VI) (eqs 10, 11, 13, and 14).^41,68^ Similarly, Fe(IV), if generated, undergoes fast bimolecular self-decay to generate inactive Fe(III) (eq 15); hence, the DET pathway could lead to a 1:1 overall stoichiometry (12, 15).
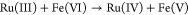
10
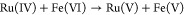
11
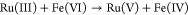
12
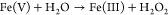
13
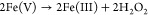
14
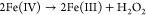
15

Therefore, the stoichiometry of the reaction between Ru(III) and ferrate(VI) may shed light on the reaction pathway between Ru(III) and ferrate(VI). Solutions of Ru(III) and ferrate(VI) were mixed at different molar ratios (0.10–2.0), and the absorbance spectrum of the mixture was recorded, as shown in Figure S4. The featured absorption peak of ferrate(VI) at 510 nm wavelength started disappearing when Ru(III) was present in the reaction mixture, and the peak at 510 nm completely disappeared at a molar ratio of 0.5. According to the spectrum, no ferrate(VI) was present in the mixed solution at the molar ratios >0.5. The stoichiometry of 1:2 suggests that each mole of Ru(III) reacted with two moles of ferrate(VI) via sequential SET transfer reactions (eqs 10 and 11) rather than one-step DET (eq 12). One may question whether the 1:2 stoichiometry could be explained by sequential DET reactions, generating Ru(VII) as the final product. However, the representative broadband absorption of Ru(VII), ranging from 400 to 700 nm, is not observed in Figure S4.^32^ Rather, two characteristic peaks near 310 and 386 nm for Ru(V) were developed after oxidation,^32^ indicating that Ru(V), instead of Ru(VII), was the final oxidation product, suggesting sequential SET reactions. The SET mechanism was further confirmed by the electrochemical identification of Ru(IV) (see a later discussion).

**Figure 5:**
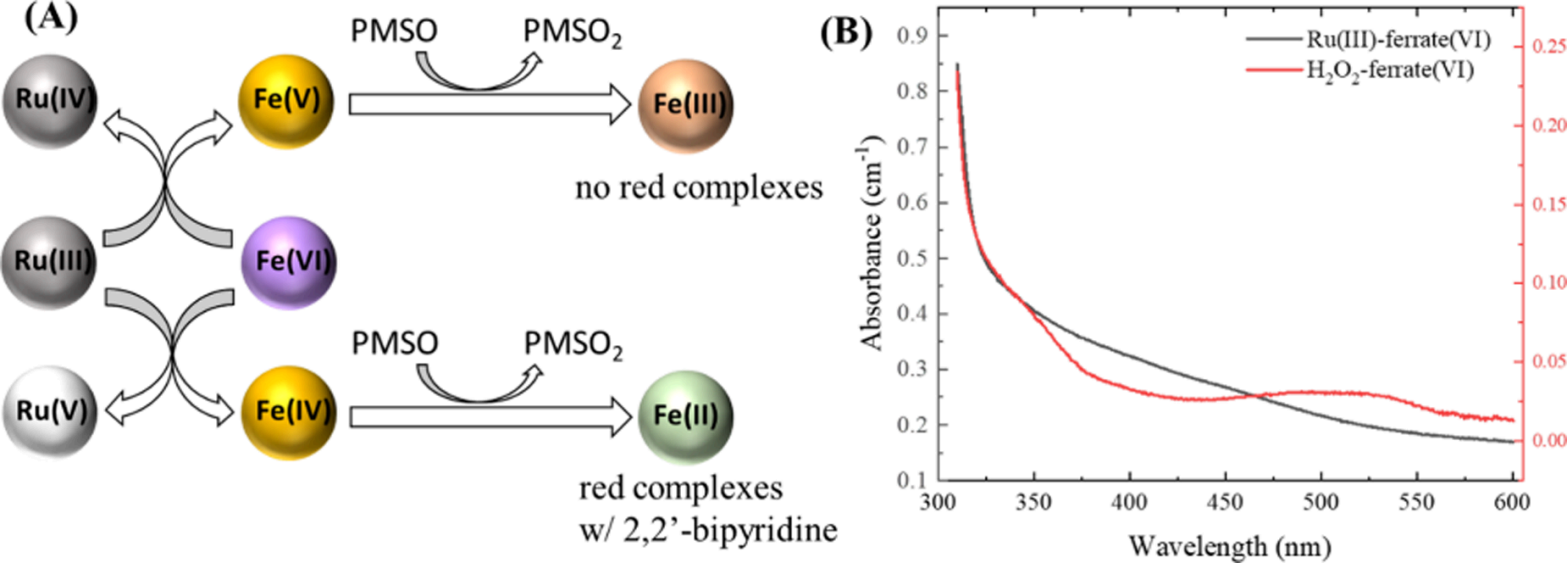
Mechanisms of the 2,2′-bipyridine method for differentiating Fe(IV)- and Fe(V)-generating pathways (A), the spectra of ferrate(VI)-H_2_O_2_ and Ru(III)-ferrate(VI) mixtures after 2.0 min reaction (B). Experimental conditions: [ferrate(VI)]_0_= 100 μM, [H_2_O_2_]_0_ = [Ru(III)]_0_ = 100 μM, [PMSO] = 50 μM, [2,2′-bipyridine] = 1 mM, pH = 8.0, [borate buffer] = 10 mM.

In addition, the reaction mechanism of Ru(III) with ferrate(VI) could be studied by tracking the formation of Fe(IV) (Figure 5A). It is well documented that PMSO reacts with Fe(IV)/Fe(V) via the DET pathway, hence generating Fe(II)/Fe(III), respectively, and Fe(II) could be detected by its complexation with 2,2′-bipyridine, which absorbs at 450–550 nm with a peak near 522 nm (ε = 8650 M^–1^ cm^–1^). Therefore, in the presence of abundant PMSO, generated Fe(IV) could be reduced to Fe(II) and results in cumulative formation of Fe(II)-2,2′-bipyridine complexes that absorb near 522 nm.^65,69^ On the contrary, Fe(V), if generated, will undergo self-decay or get reduced by PMSO, both forming Fe(III) and could not develop the complexes with 2,2′-bipyridine. As expected, the ferrate(VI)-H_2_O_2_ mixture, a well-known Fe(IV)-generating AOP (Figure 5B), exhibited a clear peak around 450–550 nm after a 2.0 min reaction. Note that, Fe(IV) generation due to the reaction between ferrate(VI) and PMSO within 2.0 min was negligible.^41^ However, Ru(III)-ferrate(VI) mixture exhibited a spectra with a broadband absorbance across 300–600 nm (Figure 5B), which was similar to that without 2,2′-bipyridine (Figure S4), while no obvious peak near 522 nm was detected. Thus, Fe(IV) generation during the 2 min reaction between Ru(III)-ferrate(VI) was negligible, suggesting that Fe(V) and high-valent Ru were major reactive species responsible for the fast decontamination in Figure 4.

Hence, Ru(III) reacted with ferrate(VI) via SET to produce Ru(IV), Fe(V), and then Ru(V). Fe(IV) did not form to react with ATL, and the short-lived Fe(V) caused the initial rapid degradation stage of the ATL in Figure 4. At molar ratios <0.5, Ru(III) would be totally oxidized to Ru(V) by ferrate(VI), hence responsible for the slower degradation stage of ATL in Figure 4. At molar ratios ≥0.5, more Ru(IV) may be accumulated as an intermediate due to incomplete oxidation of Ru and may also participate in degrading ATL.

### Electrochemical Characterization of High-Valent Ru

In previous studies, high-valent Ru has only been characterized by spectroscopic or spectrophotometric methods, but the electrochemical property of aqueous ligand-free Ru has been scarcely studied.^32,33^ In our initial experiments, the formation of stable Ru oxidant species was explored by monitoring the open circuit potential (OCP) during the reaction between Ru(III) and ferrate(VI) at different molar ratios and pH 9.0 (Figure S5). An increase in potential in OCP usually suggests that the formation of oxidative species was electrochemically detectable. At a Ru(III):Fe(VI) molar ratio of 0.10, a slow increase in the level of the OCP was observed, indicating the formation of an additional oxidative species (Figure S5A). The generation rate of the newly formed species increased as the molar ratio increased from 0.25 to 0.50 (Figure S5B,C).

**Figure 6:**
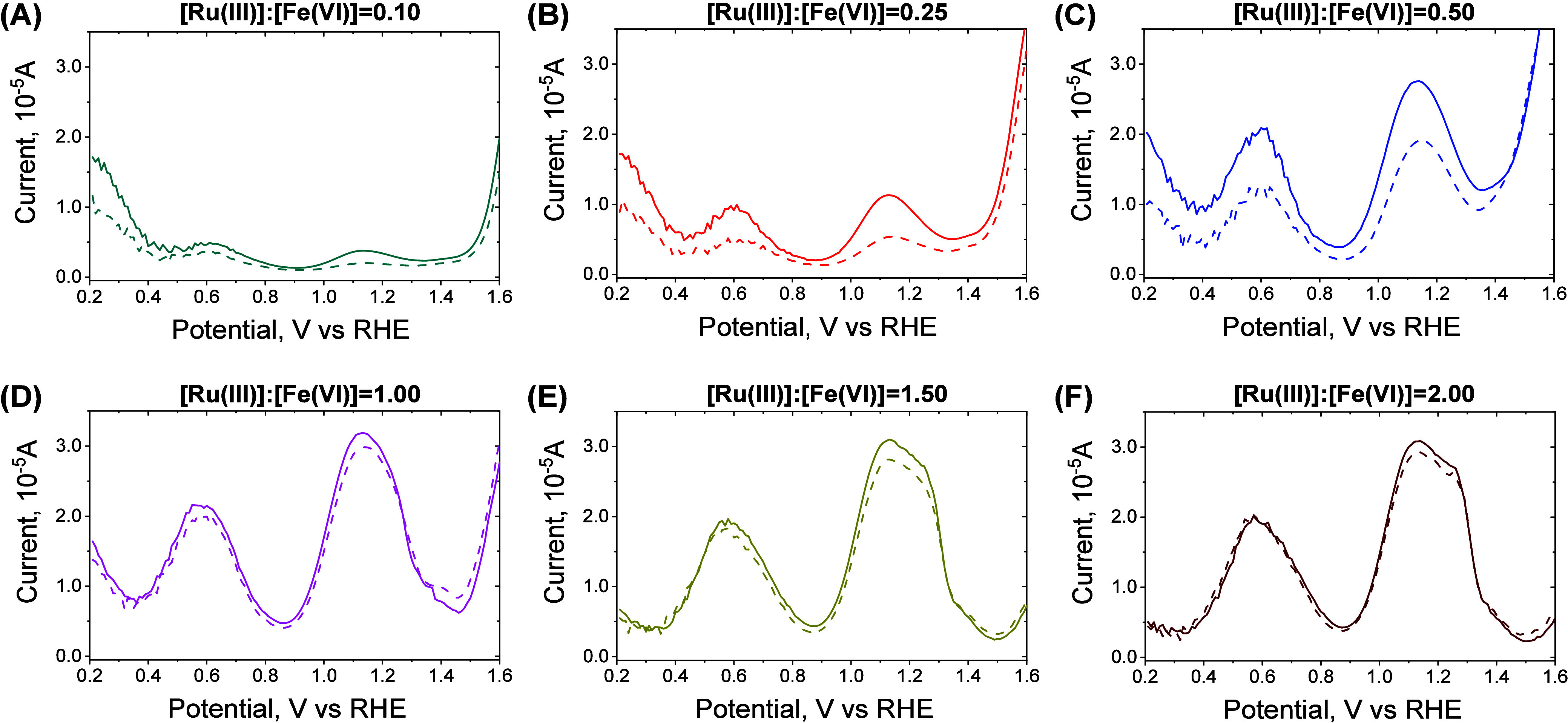
Differential pulse voltammetry (DPV) measurements of the reaction mixtures of Ru(III) and ferrate(VI) at different molar ratios. Dashed line: 30 s and solid line: 180 s. (A) 0.10, (B) 0.25, (C) 0.50, (D) 1.0, (E) 1.5, and (F) 2.0. The values of potentials are with respect to the reversible hydrogen electrode. Experimental conditions: [ferrate(VI)]_0_ = 100 μM, pH = 9.0, [borate buffer] = 10 mM.

**Figure 7:**
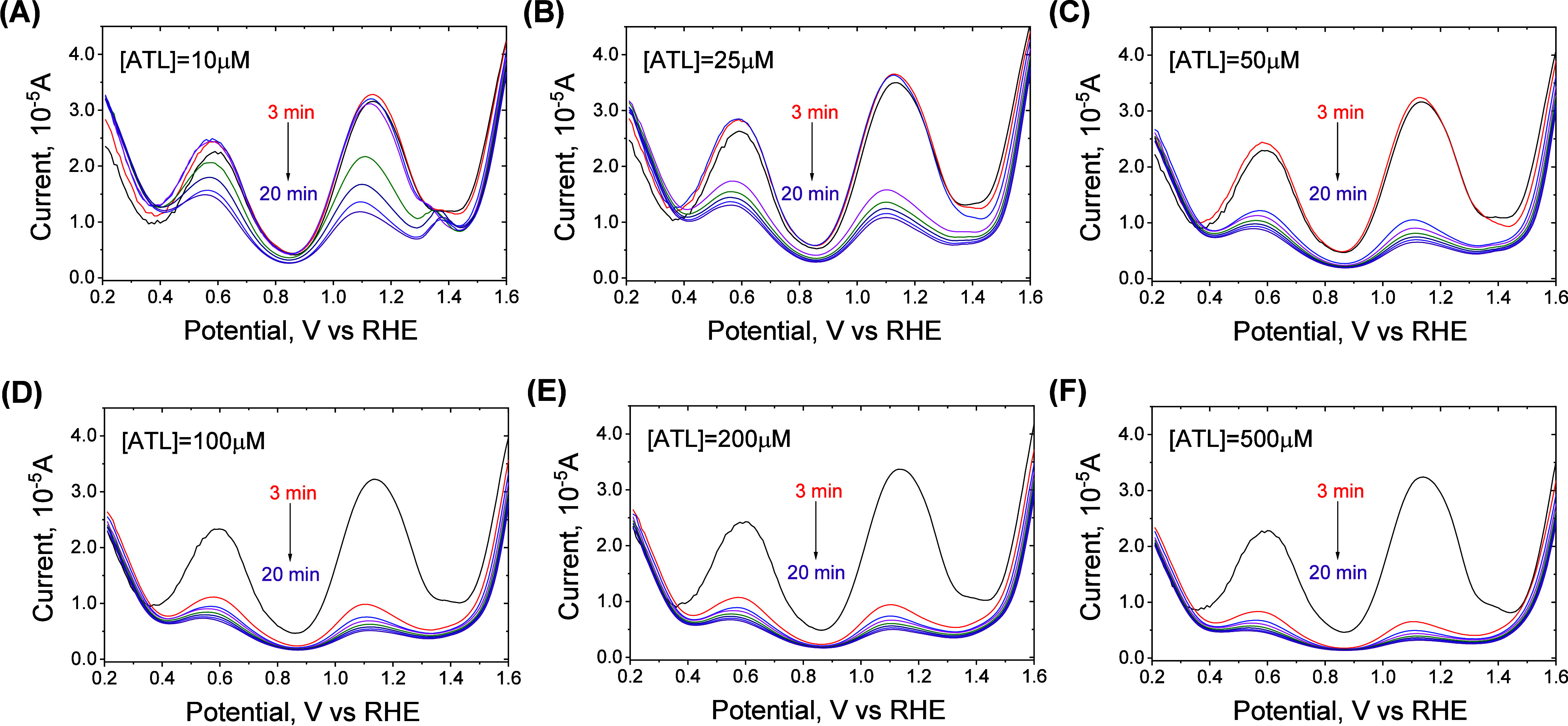
Effect of ATL addition on the differential pulse voltammetry (DPV) measurements of the mixture of Ru(III) and ferrate(VI). Experimental conditions: [ferrate(VI)]_0_ = 100 μM, pH = 9.0, [borate buffer] = 10 mM. ATL was added at 3 min, when the DPV measurement was taken. The values of potentials are with respect to the reversible hydrogen electrode.

The DPV measurements using different molar ratios of Ru(III) with ferrate(VI) were collected at 30 and 180 s (pH 9.0) and shown in Figure 6, where two peaks at ∼0.61 and ∼1.14 V appeared. These peaks corresponded to the reaction products in the Ru(III)-ferrate(VI) system, while individual Ru(III) and ferrate(VI) solutions had no such peaks (Figure S6). Notably, Fe(IV)/Fe(V) have low steady-state concentrations due to their chemical instability and, hence, could negligibly contribute to the DPV patterns. To identify the corresponding species, the peaks of Ru(IV) and Ru(V) were confirmed by conducting DPV analysis on Ru*O*_2_(*s*) and Ru(III)-PAA resultant solution (generating stable Ru(V)), respectively (Figure S7). Ru(V) generated by Ru(III)-PAA had the highest peak at ∼1.36 V (Figure S7B), which is similar to the largest peak observed in the Ru(III)-periodate (Figure S7C). Ru(IV) in the solid RuO_2_ form exhibited a peak at ∼1.14 V (Figure S7A), which is similar to the peak in Ru(III)-ferrate(VI), further confirming Ru(IV) as intermediate species during SET reactions between Ru(III) and ferrate(VI). Notably, the intensities of the 0.61 and 1.14 peaks were at the same ratio across all the experiments in different systems (Figures 6 and 7), suggesting that they represented the same species at different redox couples (e.g., *E*_Ru(IV)/Ru(III)_ and *E*_Ru(IV)/Ru(II)_). In addition, the 0.61 and 1.14 V peaks for Ru(IV) were also found in the Ru(III)-periodate system, suggesting that SET may also occur in Ru(III)-periodate, while a detailed investigation is beyond the scope of this study.

The DPV measurements were also performed at pH 8.2, which also showed similar features of the peaks (Figure S8). The growth of peaks was faster than at pH 9.0, indicating a faster reaction between Ru(III) and ferrate(VI) and concurring with the faster ATL degradation.

### Oxidation Capacity of High-Valent Ru

The oxidation of ATL by Ru(IV) was first investigated by quenching the DPV peak at ∼1.14 V by ATL. The reaction between Ru(III)-ferrate(VI) was allowed to occur for 180 s, and then ATL was added into the mixture at various concentrations (10–500 μM). The DPV curves were constructed to follow the Ru(IV) species at various time intervals (Figure 7). The decrease in current density with time at different concentrations of ATL is plotted in Figure S9. As expected, as low as 10 μM ATL was able to reduce the Ru(IV) peak at ∼1.14 V (Figure 7A), and higher ATL concentrations (100–500 μM) resulted in faster Ru(IV) consumption (Figure 7B–F).

To quantify the reactivity of Ru(IV)/Ru(V), the degradation of ATL and PMSO was tested using commercial Ru(V) preformed by Ru(III)-periodate (Figure S10). It is a well-documented DET reaction between Ru(V) and PMSO, and the second-order rate constant could be modeled by monitoring PMSO oxidation by equimolar Ru(V) preformed by Ru(III)-periodate (eqs 16–18).
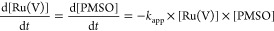
16
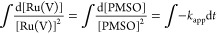
17
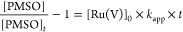
18where *t* is the reaction time (in s), [Ru(V)] and [PMSO] are the concentrations of Ru(V) and PMSO in M, *k*_app_ is the apparent second-order rate constant between Ru(V) and PMSO and was determined to be 6.7 × 10^1^ M^–1^ s^–1^ at pH 9.0 (Figure S10). This rate constant is ∼4 orders of magnitude lower than that between Fe(V) and PMSO (1.25 × 10^6^ M^–1^ s^–1^).^70^ Nonetheless, the Ru(V) is stable in the aqueous solution and hence can reach a steady-state concentration as high as 100 μM, while Fe(V) usually has a peak concentration <1 μM due to its fast self-decay (5–100 s^–1^).^65,70^ Hence, Ru(V) could provide a slower but comparable and longer degradation stage for contaminants (the second stage in Figure 4). Therefore, the initial rapid oxidation stage (Figure 4) could be attributed to the reactive and short-lived Fe(V) species, while Ru(V) was responsible for the following long-lasting oxidation stage. Fe(IV) was negligibly produced or contributed to oxidation in this system.

Unfortunately, we found that the Ru(IV) in the Ru*O*_2_(*s*) form could not react with ATL or PMSO (Figure S11), probably due to its extremely low solubility in water; hence, it may not represent the reactive Ru(IV) species formed by *in situ* Ru(III) SET oxidation, and the rate constants between Ru(IV) and ATL, PMSO remained unknown. Therefore, the rate constant between Ru(V) and ATL could not be determined because SET oxidation and Ru(IV) intermediate formation may be involved.

### Environmental Significance

In this study, we reinvestigated the reactions between Ru(III) with POAs and ferrate(VI) and revealed DET/OAT and SET pathways, respectively. Interestingly, these mechanisms differed from those in previous Ru(III) studies but were consistent with the studies on PAA and ferrate(VI) activation by other metals. Thus, far, it has been well documented that most metals, including Fe(II),^36^ Co(II) (SET may also occur, still in debate),^37,71^ and Fe(III)-complexes,^38,72,73^ react with PAA through DET reactions. On the other hand, ferrate(VI) oxidizes various metal species via SET, including Fe(II),^63,70^ Fe(III),^66^ Ag(I),^74^ and Cu(II).^75^ The underlying mechanisms could be delineated by computational chemistry methods and advanced high-valent metal chemistry.

The publication numbers of metal-AOPs have been growing remarkably over the past decade, and the electron transfer mechanism is the most fundamental knowledge of metal-AOPs that determines the reactive species in the system. The new conclusions in this study highlight the importance of investigating electron transfer mechanisms by rigorous experimental design and confirm the effectiveness of comprehensive quencher tests, stoichiometry, absorbance spectra, and electrochemistry strategies for studying relevant transient species.

## Supporting Information Available

Three supplementary texts (chemicals and analytical methods) and 11 supplementary figures (degradation of micropollutants and electrochemistry data) (PDF)
