# Supplementary material for: Revisiting the Electron Transfer Mechanisms in Ru(III)-Mediated Advanced Oxidation Processes with Peroxyacids and Ferrate(VI)
Source: Environ Sci Technol. 2024 Jun 20;58(26):11822–32. doi: 10.1021/acs.est.4c02640 (PMC11223481; doi:10.1021/acs.est.4c02640)
Supplement: Supplementary file 1 — es4c02640_si_001.pdf [file es4c02640_si_001.pdf]

Supporting Information

Revisiting the Electron Transfer Mechanisms in Ru(III)-Mediated Advanced Oxidation Processes with Peroxyacids and Ferrate(VI)

Krishnamoorthy Sathiyan,[1,≠] Junyue Wang,[2,≠] Lois M. Williams,[1]

Ching-Hua Huang[2*], and Virender K. Sharma[1*]

[1]Program for Environment and Sustainability, Department of Environmental and Occupational Health, School of Public Health, Texas A&M University, College Station, Texas, 77843-8371, USA, vsharma@tamu.edu

[2]School of Civil and Environmental Engineering, Georgia Institute of Technology, Atlanta, GA 30332, USA, ching-hua.huang@ce.gatech.edu

[≠]The authors contributed equally to this work.

*Corresponding Authors.

E-mails: vsharma@tamu.edu (Virender K. Sharma);

ching-hua.huang@ce.gatech.edu (Ching-Hua Huang)

Numbers of Pages: 13

Numbers of Figures: 11

Numbers of Texts: 1

Text S1. Chemicals

Ruthenium chloride ($RuCl_3 \bullet H_2O$), ruthenium dioxide (RuO_2), sodium periodate ($NaIO_4$), atenolol (ATL), sulfamethoxazole (SMX), naproxen (NPX), carbamazepine (CBZ), trimethoprim (TMP), sulfamethoxazole (SMX), aspartame (APT), diatrizoic acid (DTA), methyl phenyl sulfoxide (PMSO), methyl phenyl sulfone ($PMSO_2$), tert-butyl alcohol (TBA), 2,4-hexadiene, 2,2'-bipyridine, sodium hydroxide (NaOH), sulfuric acid (H_2SO_4), sodium thiosulfate ($Na_2S_2O_3$), acetate ($CH_3C(O)OH$), potassium iodide (KI), N,Ndiethyl-p-phenylenediamine (DPD), 2,2'-azino-bis(3-ethylbenzothiazoline-6-sulfonic)acid (ABTS), sodium chloride (NaCl), sodium tetraborate ($Na_2B_4O_7 \bullet 10H_2O$), and sodium hydrogen phosphate were purchased from Sigma-Aldrich or Fisher Scientific (Fair Lawn, NJ) at the highest avaiable purity. Deionized water (DI water) (> 18 mΩ-cm) was produced from a Milli-Q water purification system (Billerica, MA).

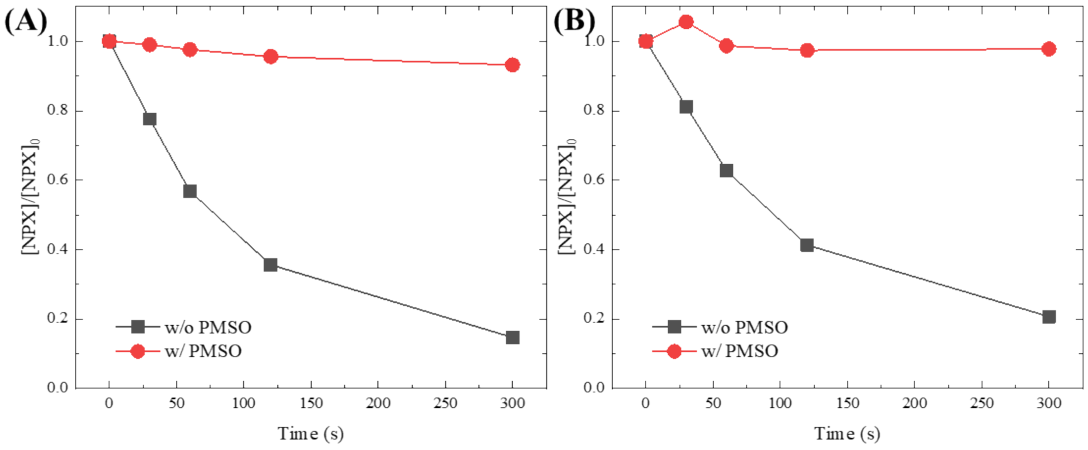

Figure S1. Degradation of NPX by Ru(III)-PAA (A) and Ru(III)-PFA (B) processes with and without PMSO. Experimental conditions: $[NPX]_0$ = 5 μM, $[PMSO]_0$ = 1 mM, $[POAs]_0$ = $[Ru(III)]_0$ = 100 μM, pH = 7.0, [phosphate buffer] = 10 mM.

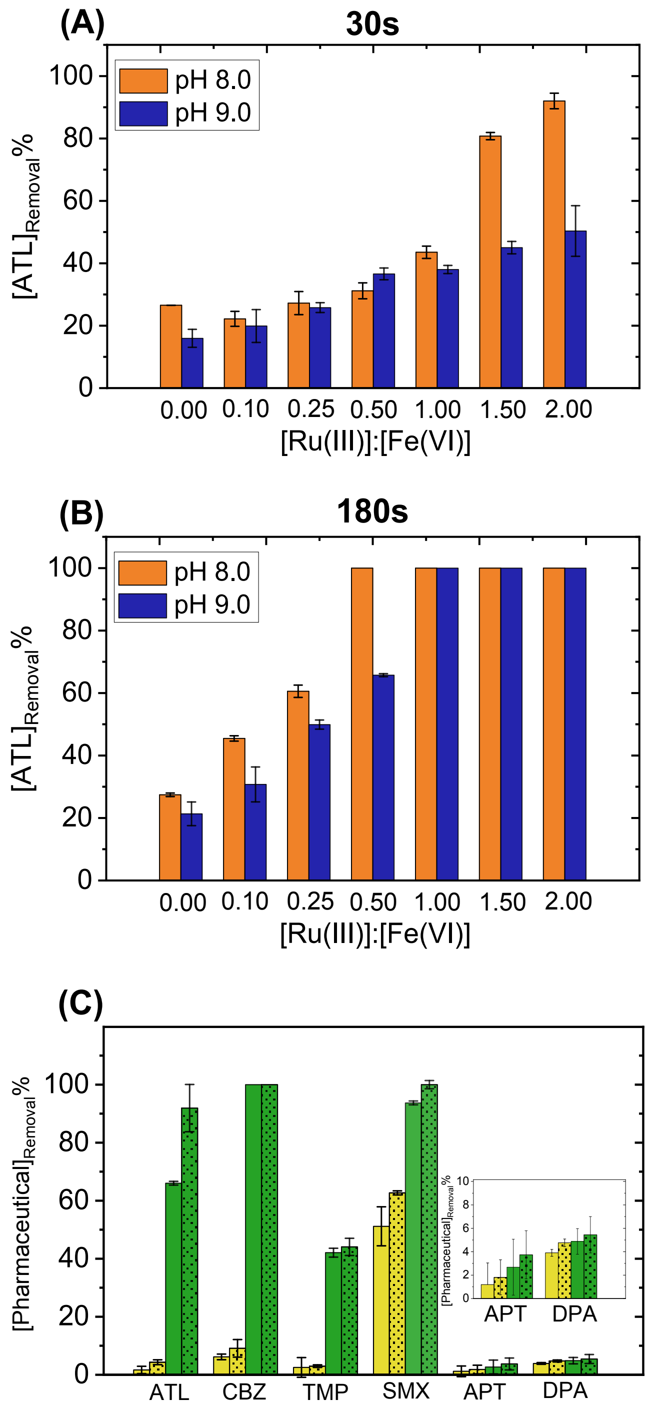

Figure S2. (A) Effect of molar ratio of Ru(III) to Ferrate(VI) in with various concentrations at different pHs, pH 8.0 (orange) and pH 9.0 (blue) at (A) 30 s and, (B) 180 s, and (C) removal of different micropollutants at pH 8.0 at reaction time 90 s (empty column) and 180 s (filled column) (yellow represent ferrate(VI) alone and green represents [Ru(III)]:[ferrate(VI)]=0.50). Experimental conditions: [contaminants] = 5 μM, $[Ru(III)]_0$ = 50 μM, $[ferrate(VI)]_0$ = 100 μM, [borate buffer] = 10 mM.

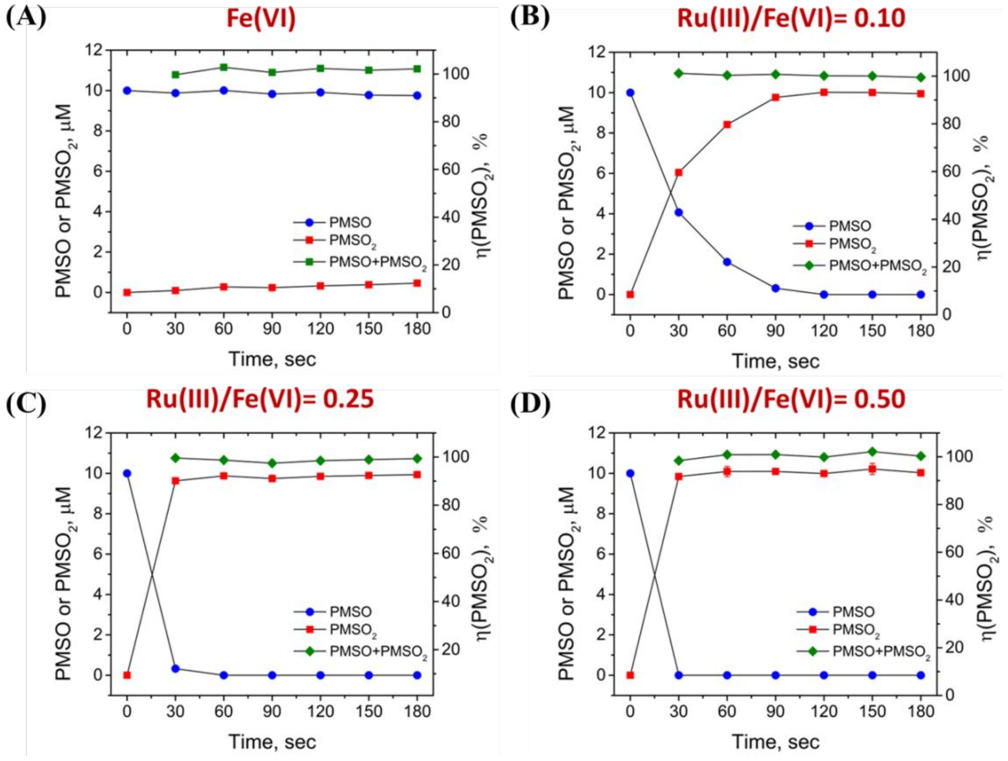

Figure S3. PMSO oxidation and $PMSO_2$ formation by Ru(III)-ferrate(VI). Experimental conditions: $[ferrate(VI)]_0$= 100 μM, pH = 9.0, [borate buffer] = 10 mM.

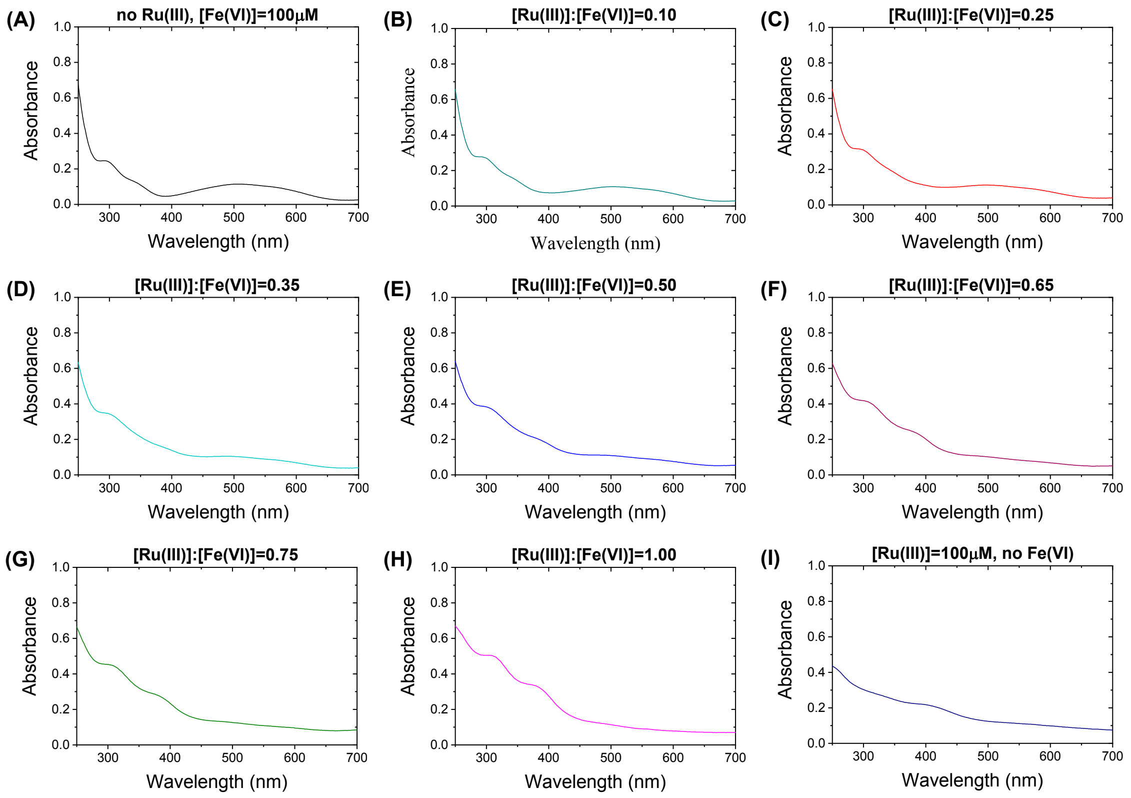

Figure S4. The absorbance spectra of Ru(III)-ferrate(VI) mixture. Experimental conditions: [ferrate(VI)]$_0$= 100 μM, pH = 9.0, [borate buffer] = 10 mM.

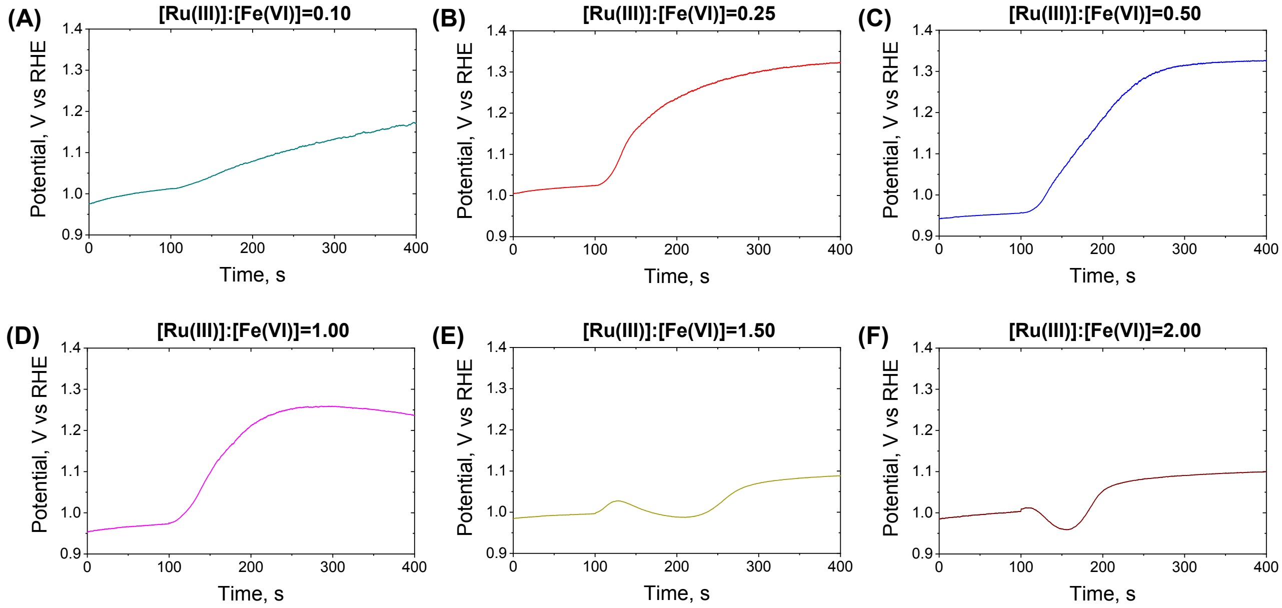

Figure S5. Open Circuit Potential (OCP) with time measured for different concentrations of Ru(III)-ferrate(VI) system. Experimental conditions: Ru(III) = 10-200 μM, ferrate(VI) = 100 μM, pH 9.0, [borate buffer] = 10 mM; Ru(III) was added at 100 s.

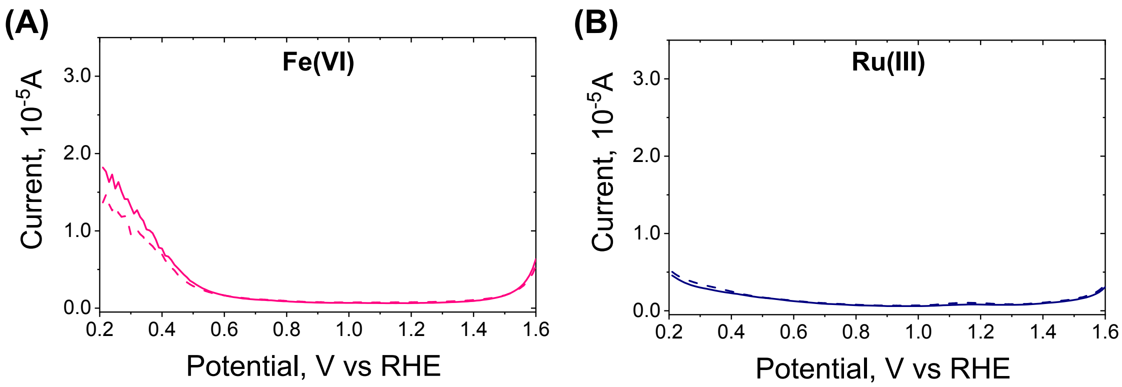

Figure S6. DVP measurements of ferrate(VI) and Ru(III) (Dashed line: 30 s and solid line: 180 s). The potentials are with respect to reversible hydrogen electrode. Experimental conditions: [Ru(III)] = 100 μM, [ferrate(VI)] = 100 μM, [borate buffer] = 10 mM, pH 9.0.

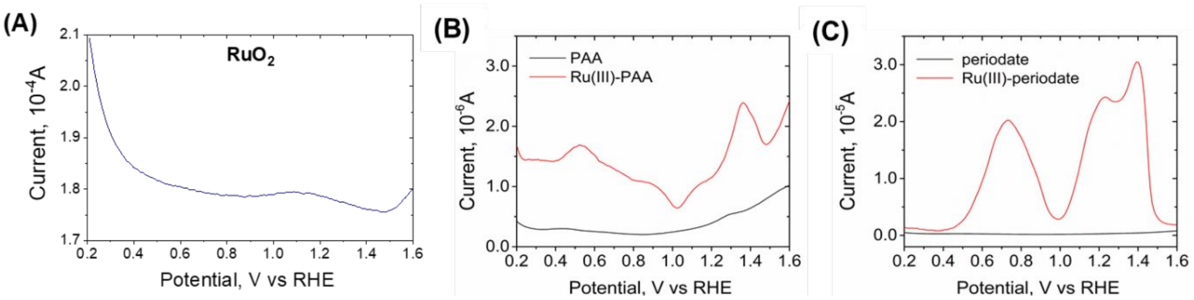

Figure S7. Differential pulse voltammetry (DVP) measurements of RuO_2(s) (A), Ru(III)-PAA (B), and Ru(III)-periodate (C). Experimental conditions: $[Ru(III)]_0 = [periodate]_0 = [PAA]_0 = 100\ \mu M$, pH = 7.0, phosphate buffer = 10 mM, reaction time = 60 s for B, C.

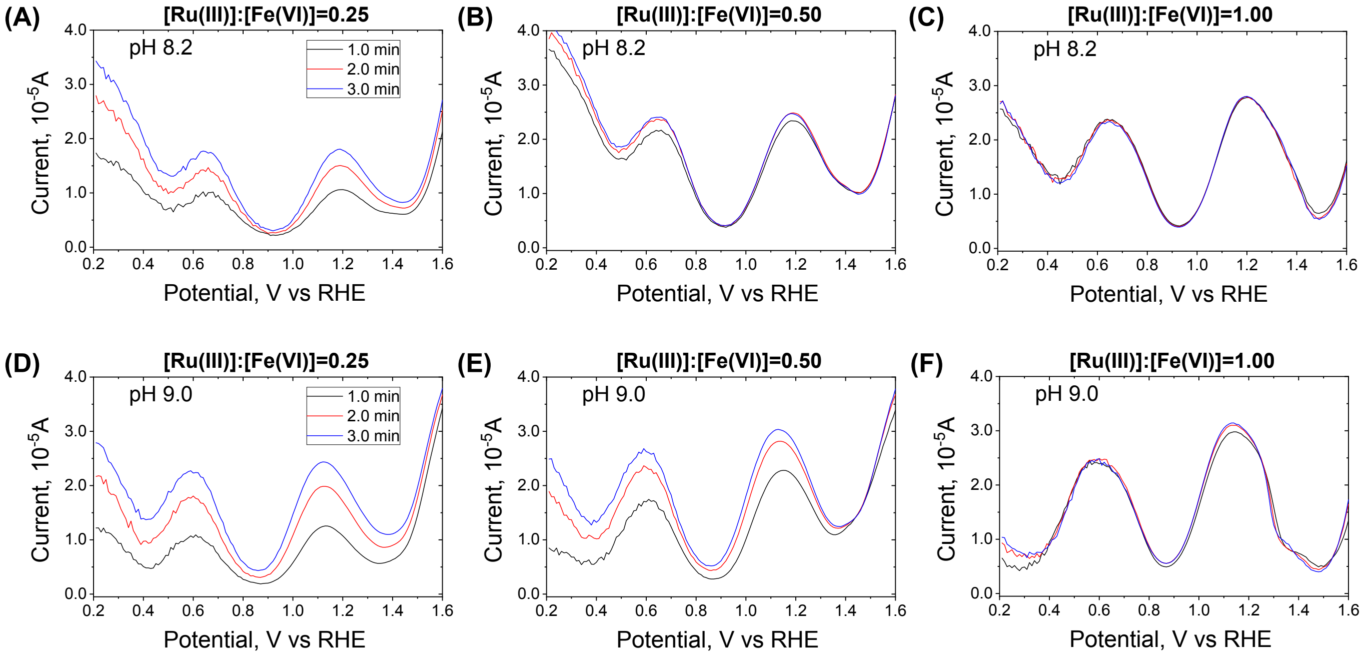

Figure S8. Differential pulse voltammetry (DPV) measurements at different molar ratios at pH 8.2 and pH 9.0. The potential mentioned herein are with respect to reversible hydrogen electrode. Experimental conditions: [ferrate(VI)]$_0$ = 100 µM, borate buffer = 10 mM, scan rate = 50 mV s^{-1}.

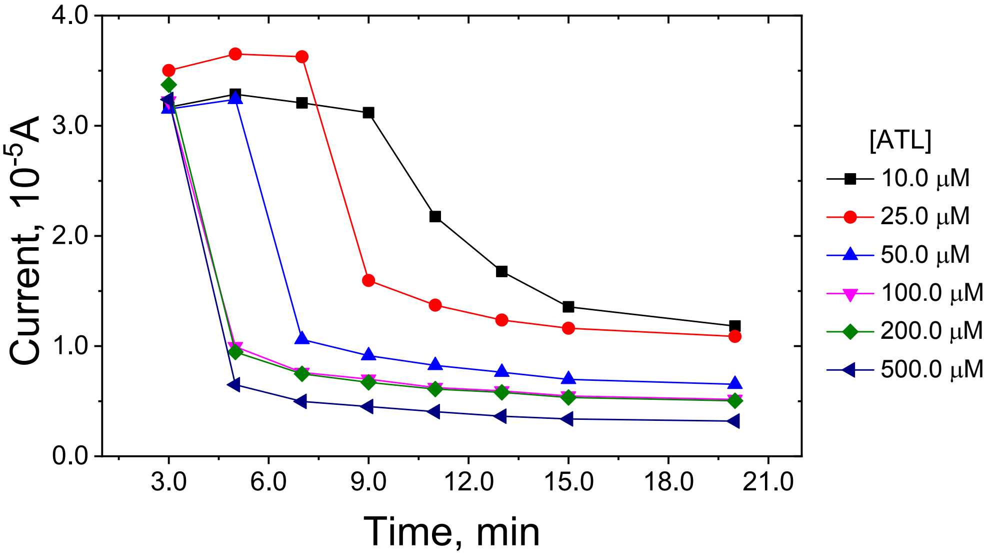

Figure S9. Plot showing the current vs time from DPV with different concentration of ATL in after 3-min reaction with Ru(III) and ferrate(VI). Experimental conditions: $[\text{ferrate(VI)}]_0$ = 100 µM, $[\text{Ru(III)}]_0$ = 50 µM, borate buffer = 10 mM, scan rate = 50 mV s^{-1}.

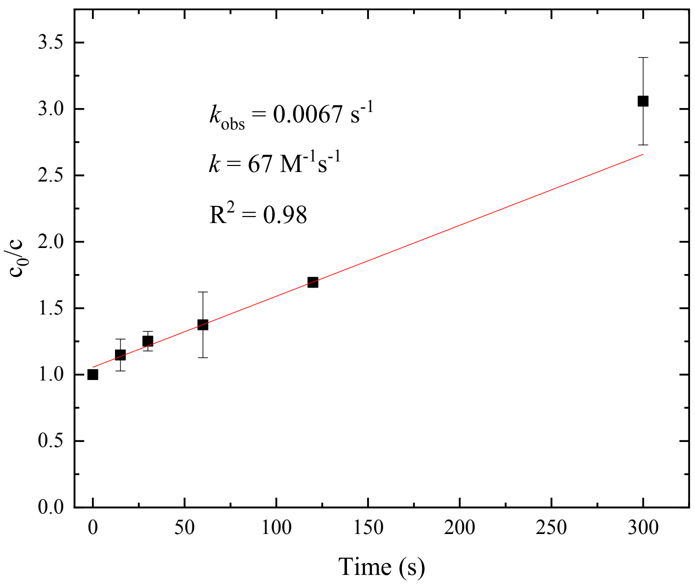

Figure S10. Degradation of PMSO by Ru(V). Experimental conditions: $[Ru(V)]_0$ = 100 μM (generated by Ru(III)-periodate), [PMSO] = 100 μM, pH = 9.0, [borate buffer] = 10 mM.

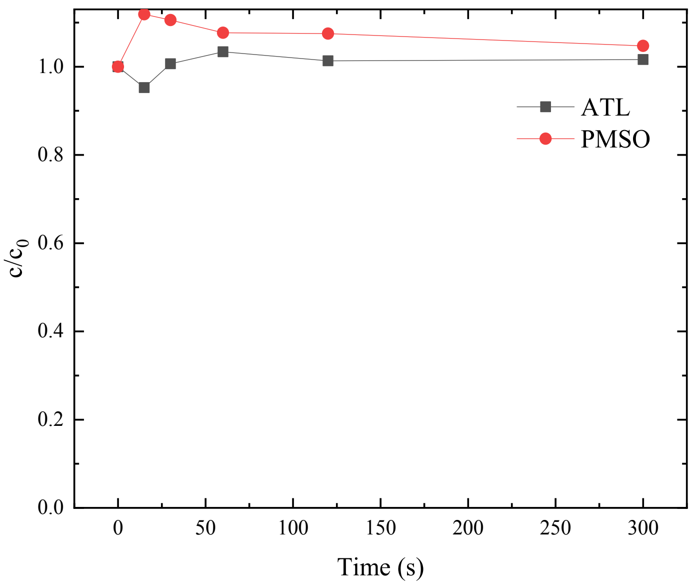

Figure S11. Degradation of ATL and PMSO by RuO_2. Experimental conditions: $[RuO_2]_0$ = 100 μM, $[PMSO]_0 = [ATL]_0$ = 5 μM, pH = 9.0, [borate buffer] = 10.0 mM.
